# An open and georeferenced dataset of forest structural attributes and microhabitats in central and southern Apennines (Italy)

**DOI:** 10.1016/j.dib.2022.108445

**Published:** 2022-07-08

**Authors:** Francesco Parisi, Saverio Francini, Costanza Borghi, Gherardo Chirici

**Affiliations:** a Department of Agriculture, GeoLAB - Laboratory of Forest Geomatics Food, Environment and Forestry, Università degli Studi di Firenze, Firenze, Italy; b Fondazione Per il Futuro delle Città, Firenze, Italy

**Keywords:** Forest ecosystems, Mediterranean mountains, Ecological indicators, Broadleaved mixed forest, Silver fir, Beech, Chestnut

## Abstract

Forests cover 30% of the Earth's landmass, host 80% of the biodiversity on land, and represent one of the main sinks of carbon. Studying forest ecosystems and dynamics is more crucial than ever now that the climate is changing. On the other hand, forest structural attributes and microhabitats data acquisition is challenging, and require huge efforts.

Here we provide a georeferenced dataset of living trees, deadwood, and microhabitats referring to 199 plots (13 m radius), collected between 2012 and 2018, and located over six Apennine mountainous forest types across Italy. The dataset we provide promotes collaboration among researchers and improves the possibilities to analyze the evolution of forest ecosystems.

## Specifications Table

**Table:** 

Subject	Agricultural science: Forestry
Specific subject area	Dendrometric measurement; Living trees, deadwood, and microhabitats survey
Type of data	Geospatial point vector (shapefile)Table
How the data were acquired	The dataset was constructed through field measurements of 199 sample plots (radius 13 m) distributed in six Italian forests (Table 1. Living trees, deadwood, and microhabitats data were acquired through the application of specific protocols. The instruments we used to acquire data include a tree caliper, GPS systems, hypsometry, and a vertex.
Data format	RawAnalyzed
Description of data collection	Fieldwork was performed for each of the 199 georeferenced sites to acquire data related to (i) the number and the volume of living trees, (ii) the volume of deadwood, and (iii) types and abundance of tree-related microhabitats on living and deadwood. The information is provided both at site and tree levels.
Data source location	Four Italian administrative regions (NUT2): Abruzzo, Molise, Campania and Calabria
Data accessibility	Repository name: Mendeley DataData identification number: 10.17632/nwsw7hvn5t.1Direct URL to data: https://data.mendeley.com/datasets/nwsw7hvn5t/1

**Value of the Data**

• This dataset can be used to analyze forest stand structures and microhabitat typologies in different Apennine mountainous forest types.
• We want to promote collaboration among researchers by making datasets available, this dataset will assist forest researchers to collaborate as well as to combine and extend their data for further analysis.
• This dataset will improve the possibility for forest researchers and managers of analyzing the evolution of forest ecosystems for long-term studies.
• We encourage repeating forest assessments in the same localities to evaluate the trends in ecological indicators over time and space.
• If integrated with the dataset we provided in Campanaro and Parisi [1], these data can be used to investigate the relationship between saproxylic and non-saproxylic beetle communities and forest structural attributes.

## 1 Data Description

We provide two different datasets: one at the plot level (both .xlsx and .shp formats) and one at the tree level (xlsx format). While the coordinates of each plot are available - and that is why we provide a shapefile for plots – the coordinates of each tree were not acquired. The description and the measuring unit of all the attributes included in the dataset are provided in Table 1. Furthermore, in some sites specific projects are underway, and not all the forest attributes are available (e.g., forest structure in the Matese area). Table 1 also includes information regarding the availability of data. The plot-level dataset contains 199 rows, one row for each of 199 sample plots located in central and southern Italy over six different regions, for which a detailed description is provided in the next section.

Tables 2 and 3 describe the dataset related to single-tree data, acquired in the six forest sites reported above. In particular, Table 2 provides information on the structural attributes, while Table 3 defines the tree-related microhabitat acquired at the plot level.

**Table 1:** Forest attributes and tree-related microhabitats per site description.

Variable	Description	Measure unit	Site availability
site	name of the site	-	GS, AS, BP, MT, CI, AM
acronym	acronym of the siteGS: Gran SassoAS: Abeti SopraniBP: Bosco PennataroMT: MateseCI: CilentoAM: Aspromonte	-	GS, AS, BP, MT, CI, AM
plot_ID	identification number for each plot, per site. It is composed as “*acronym*_*number of the plot”*	-	GS, AS, BP, MT, CI, AM
elevation	elevation of the plot	m a.s.l.	GS, AS, BP, MT, CI, AM
x	Coordinates of the plot centre, longitude	Standard UTM coordinates (WGS 84 33N - EPSG: 32633)	GS, AS, BP, MT, CI, AM
y	Coordinates of the plot centre, latitude	Standard UTM coordinates (WGS 84 33N -EPSG: 32633)	GS, AS, BP, MT, CI, AM
management	managed/unmanaged/orchard	-	GS, AS, BP, MT, CI, AM
canopy_cov	canopy cover for each plot	%	GS, AS, BP, CI, AM
n_trees	number of living trees per hectare	-	GS, AS, BP, CI, AM
V_trees	volume of living trees per hectare	m^3^/ha	GS, AS, BP, CI, AM
BA	basal area per hectare	m^2^/ha	GS, AS, BP, CI, AM
n_CWD	number of coarse woody debris per hectare	-	GS, AS, BP, MT, CI, AM
V_CWD	volume of coarse woody debris per hectare	m^3^/ha	GS, AS, BP, MT, CI, AM
n_SDT	number of standing dead trees per hectare	-	GS, AS, BP, MT, CI, AM
V_SDT	volume of standing dead trees per hectare	m^3^/ha	GS, AS, BP, MT, CI, AM
n_Stumps	number of stumps per hectare	-	GS, AS, BP, MT, CI, AM
V_Stumps	volume of stumps per hectare	m^3^/ha	GS, AS, BP, MT, CI, AM
n_Snags	number of snags per hectare	-	GS, AS, BP, MT, CI, AM
V_Snags	volume of snags per hectare	m^3^/ha	GS, AS, BP, MT, CI, AM
n_DDT	number of dead downed trees per hectare	-	GS, AS, BP, MT, CI, AM
V_DDT	volume of dead downed trees per hectare	m^3^/ha	GS, AS, BP, MT, CI, AM
n_mh_alive	Per plot number of per hectare typologies of tree-related microhabitats sampled on living trees.	-	AS, BP, AM*
n_mh_dead	Per plot number of per hectare typologies of tree-related microhabitats sampled on dead trees.	-	AS, BP, MT, AM*
n_mh_tot	sum of n_mh_alive and n_mh_dead	-	GS⁎⁎, AS, BP, MT, CI⁎⁎, AM*

Note:

⁎ in AM, data on tree-related microhabitats is available only per ``orchard'' management.

⁎⁎ in GS and CI, data on tree-related microhabitats is not distinguished between living and dead trees.

**Table 2:** Tree-level database description.

(Sheet 1) Forest structure		(Sheet 4) Stumps	
Head	Description	Head	Description
site	Name of the site	acronym	Acronym of the site
acronym	Acronym of the site	plot_ID	Number of the plot, per each site
N2k_CDDA	Natura 2000 or Nationally designated areas (CDDA) code	ID_stump	Identification number per each SDT in each plot
plot_ID	Number of the plot, per each site	Origin (N/A)	Origin of the stump, Natural or Artificial
man_type	type of forest management	Dbase_stump (cm)	base diameter of each stump
man_regime	regime of forest management	Dtop_stump (cm)	top diameter of each stump
EEA_type	forest type according to European classification [2]	h_stump(m)	height of each stump
ID_tree	Identification number per CWD in each plot	Sp_stump	Species of the stumps
sp_tree	Tree species (latin name)	decay_stump	decay stage [3]
dbh_tree (cm)	diameter at breast height	V_stump(m3)	volume of each stump in each plot
		
h_tree (m)	Tree height	Sheet 5) Snags	
V_tree (m3)	Tree volume	acronym	Acronym of the site
BA_tree (m2)	Tree basal area	plot_ID	Number of the plot, per each site
		
Sheet 2) CWD - Coarse Woody Debris		ID_snag	Identification number per each snag in each plot
acronym	Acronym of the site	Dtop_snag(cm)	top diameter of each snag in each plot
plot_ID	Number of the plot, per each site	Dbase_snag(cm)	top diameter of each snag in each plot
ID_CWD	Identification number per each CWD in each plot	h_snag(m)	height of each snag in each plot
Dmin_CWD (cm)	minimum diameter of the CWD	Sp_snag	specie of each snag in each plot
Dmax_CWD (cm)	maximum diameter of the CWD	decay_snag	decay stage [3]
lenght_CWD (m)	Length of the CWD	V_snag(m3)	volume of each snag in each plot
		
sp_CWD	species of the CWD	Sheet 6) DDT - Dead Downed Trees	
decay_CWD	decay stage [3]	acronym	Acronym of the site
V_CWD (m3)	Volume of the CWD	plot_ID	Number of the plot, per each site
		
Sheet 3) SDT - Standing Dead Tree		ID_DDT	identification number of each DDT in each plot
acronym	Acronym of the site	dbh_DDT (cm)	diameter at breast height of each DDT in each plot
plot_ID	Number of the plot, per each site	lenght_DDT (m)	Length of each DDT in each plot
ID_SDT	Identification number per each SDT in each plot	Sp_DDT	specie of each DDT in each plot
dbh_SDT (cm)	diameter at breast height per each stump in each plot	decay_DDT	decay stage [3]
h_SDT (m)	height per each SDT in each plot	V_DDT (m3)	volume of each DDT in each plot
sp_SDT	tree species per each SDT in each plot		
decay_SDT	decay stage [3]		
V_SDT (m3)	volume per each SDT in each plot

**Table 3:** Tree-related microhabitat definitions.

(Sheet 7) MW Tree-related microhabitats [4,5]		(Sheet 8) ML Tree-related microhabitats [6,7]	
Head	Definition	Head	Definition
MW_1	Occurrence of fruiting bodies of Fomes fomentarius	ML_1	Woodpecker breeding cavities
MW_2	Occurrence of fruiting bodies of Fomitopsis pinicola	ML_2	Rot holes
MW_3	Occurrence of other fungal infection	ML_3	Concavities
MW_4	Crown broken <50%	ML_4	Insect galleries and bore holes
MW_5	Several main branches are broken: >50% of the crown broken	ML_5	Exposed sapwood only
MW_6	Broken fork: complete fracture of one of the two forking branches	ML_6	Exposed sapwood and heartwood
MW_7	Broken stem: the crown is totally absent and very small living twigs have remained	ML_7	Crown deadwood
MW_8	Substitute or secondary crown	ML_8	Twig tangles
MW_9	Lightning scar at least 3 m long and reaching the sapwood	ML_9	Burrs and cankers
MW_10	Crack: cleft into the sapwood >50cm long along the stem and at least 2 cm deep	ML_10	Perennial fungal fruiting bodies (life span> 1y)
MW_11	Splintered stem: the split-up results in numerous scales of wood >50 cm long	ML_11	Ephemeral fungal fruiting bodies and slime moulds
MW_12	Cavities with >5 cm aperture	ML_12	Epiphytic or parasitic crypto- and phanerogams
MW_13	Cavity string: at least three woodpecker cavities	ML_13	Nests
MW_14	Deep stem cavities: a tubular cavity with little or without mould	ML_14	Fresh exudates
MW_15	Cavities with mould of at least 8000 cm3	ML_15	Microsoils
MW_16	Mould pockets: space between loose bark and the sapwood		
MW_17	Bark pockets: same structure as M16, but without mould		
MW_18	Canker: proliferation of cell growth at least 10 cm in diameter		
MW_19	Bark loss: patches with bark loss of at least 5 cm caused by natural falling of trees		
MW_20	Uprooted stump, with a minimum height of 1.20 m of the vertical root plate		
MW_21	System of gallery of Scolytidae insects		
MW_22	Saproxylic insect holes		
MW_23	Water filled rot hole on stump		

## 2 Experimental Design, Materials and Methods

### 2.1 Forest Landscapes in the Dataset

This dataset refers to six study areas, characterized by forest landscapes with different characteristics in terms of both geomorphological conditions (Table 4) and management (Fig. 2). Detailed information on these study areas is reported in Campanaro and Parisi [1], for which a summary is provided below.

**Table 4:** Details of the six forest sites in the dataset.

Site	Gran Sasso	Abeti Soprani	Bosco Pennataro	Matese	Cilento	Aspromonte
Acronym	GS	AS	BP	MT	CI	AM
Municipality (study area)	Pietracamela (TE)	Pescopennataro (IS)	Vastogirardi (IS)	Roccamandolfi (IS)	Corleto Monforte (SA)	Santo Stefano (RC)
Coordinates N (decimals)	42.5096 N	41.860833 N	41.748889 N	41.452222 N	40.4705 N	38.18 N
Coordinates E (decimals)	13.5679 E	14.293611 E	14.197222 E	14.350278 E	15.4317 E	15.784167 E
Altitude (m a.s.l.)	1500	1450	1100	1700	1250	1059
Number of sampling plots	19	50	50	60	14	6
European forest type [2]	Apennine-Corsican mountainous beech forest (6.7.3)	Apennine-Corsican mountainous beech forest (6.7.3)	Sessile oak-hornbeam forest (6.5.2)	Apennine-Corsican mountainous beech forest (6.7.3)	Apennine-Corsican mountainous beech forest (6.7.3)	Chestnut forest (6.8.7)
Management regime	old high forest	old high forest	high forest on old coppice	mature coppice with standard; group system (high forest)	old high forest	mature coppice with standard; orchard; young coppice with standards

From North to South, the first site is the Gran Sasso (about 70ha), which is located in the central Apennines. It is representative of the European forest type 6.7.3, “Apennine-Corsican mountainous beech forest” [2], with a dominant height of 27.73 m. Data collection in Gran Sasso was carried out in 2013.

Second, the Abeti Soprani experimental area covers 240 ha. This forest is an almost pure *A. alba* stand, associated with *Fagus sylvatica* L. at the highest altitudes, and with *Quercus cerris* L. at the lowest altitudes. The dominant height of the sampled stands is 25.38 m, while the average age is 120–130 years; the data was collected in 2012.

Third, the Bosco Pennataro is a broadleaved mixed forest (European forest type 6.5.2) located in the Molise administrative region, covering a surface of almost 300 ha (data collection year 2014). With a dominant height of 29.20 m, the forest is characterized by a mixture of old coppices, and patches of mature forest stands grown mainly from seeds. Further, the Bosco Pennataro forest is dominated by large and tall mature trees with a closed canopy.

Fourth, the Matese forest is an Apennine beech forest with *Taxus* and *Ilex* (European forest type 6.7.3). Data collection in Matese forest was carried out in 2018. As Bosco Pennataro, Matese forest is located in the Molise administrative region and covers almost 400 ha of the Roccamadolfi forests, which is included within the Special Areas of Conservation (SAC) (http://natura2000.eea.europa.eu) "La Gallinola - Monte Miletto - Monti del Matese" (Cod. IT 7222287), within the National Park of Matese.

Then, the Cilento site (about 70 ha), which is located in the southern Apennines, is representative of montane coniferous forests (prevalent European forest type 6.7.3); the dominant height of the forest is 25.75 m, and the data was collected in 2013.

**Fig. 1:**
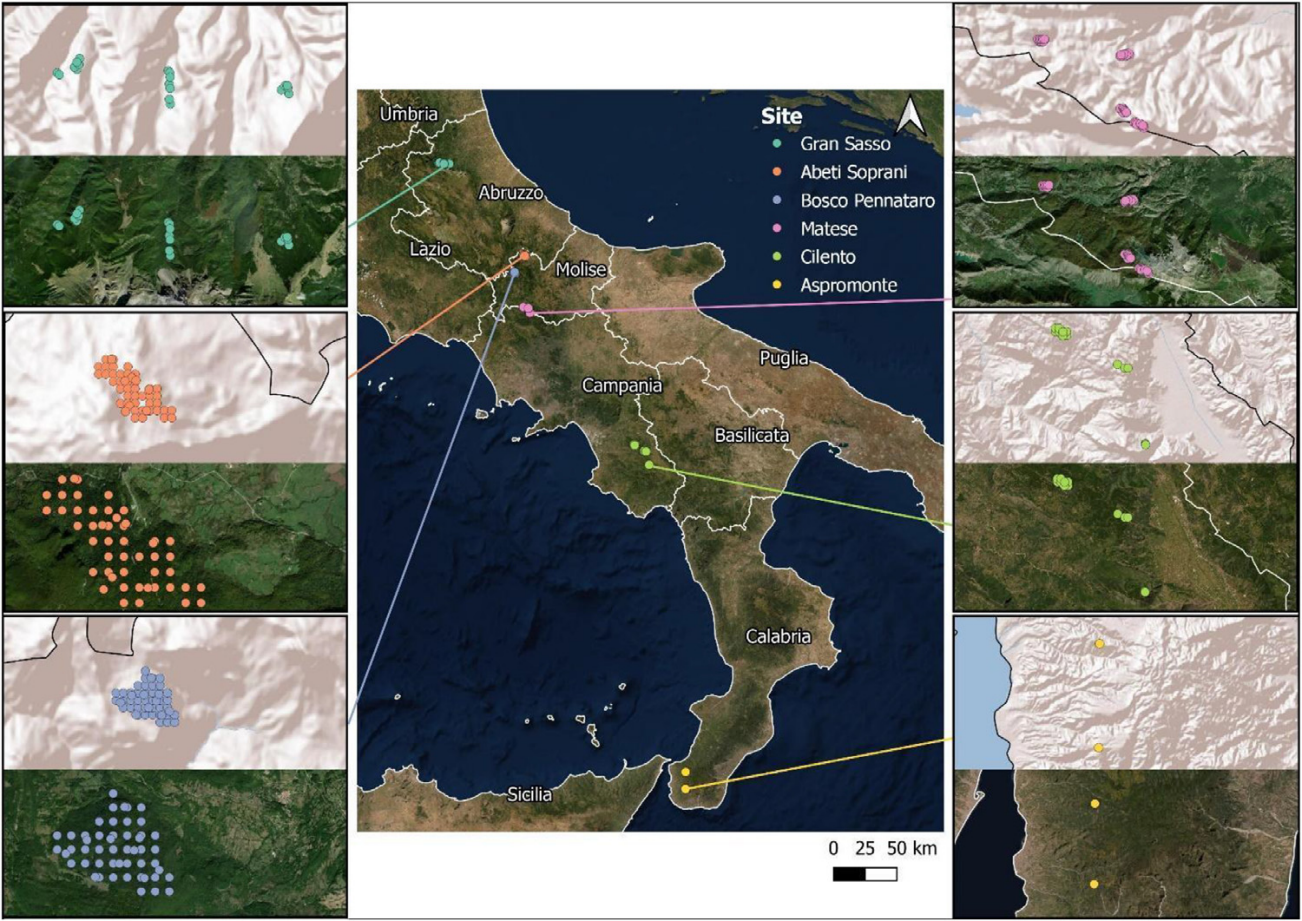
Location of the sampling plots.

Last, the Aspromonte site includes three different agroforestry systems dominated by chestnuts (European forest type 6.8.7), i.e., (i) young (2 years) and (ii) mature (11 years) coppices stands, and (iii) traditional fruit orchard (older than 80 years). These agroforestry systems are characterized by a dominant height of 11.34 m. Each of the analyzed management types extend for about 12 ha. In this site, data collection was carried out in 2017 (Fig. 1).

**Fig. 2:**
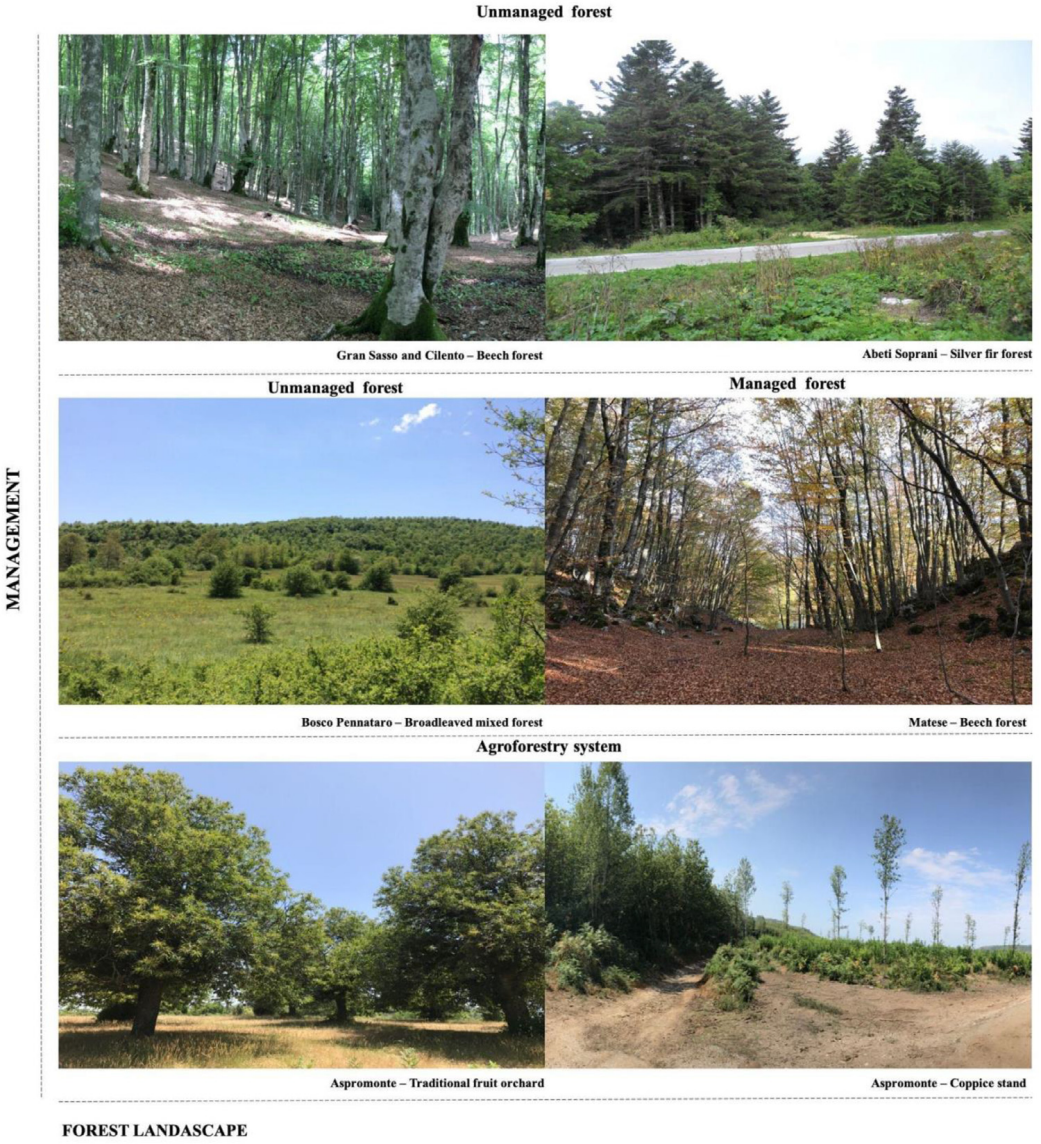
Management and forest landscapes in the six sites.

### 2.2 Data Acquisition

In each site, data were acquired on circular plots of 13 m of radius, located throughout different landscapes with diverse forest types. All sites followed a systematic aligned sampling scheme except for Gran Sasso and Cilento, where a systematic non-aligned sampling method was applied. For each sampling station, UTM datum WGS84 33N (EPGS 32633) coordinates and altitude were recorded using the Juno SB Global Positioning System. Living trees (minimum diameter at breast height, DBH, ≥ 10 cm) and deadwood (minimum diameter ≥5 cm) were measured, marked, and numbered. The information recorded on the plots comprised tree DBH and height, canopy cover (through visual estimation), and tree species (both for living and dead trees). Furthermore, dead downed trees, snags, coarse woody debris, and stumps were included in the survey, measuring their lengths, heights, and minimum and maximum diameters. Snags were defined as standing dead trees, without crowns, with a minimum height of 1.3 m [3], while standing dead trees were characterized by the presence of crowns (dead branches and twigs) [3]. The volume of living trees, standing, and dead downed trees were calculated by the double-entry volume equation [7], while the volumes of snags, coarse woody debris, and stumps were calculated through the cone trunk formula [8]. The sampling protocol used refers to the one proposed in Burrascano et al. [9].

## Ethics Statements

The authors declare that the present work did not include experiments on human subjects and/or animals.

## CRediT authorship contribution statement

**Francesco Parisi:** Data curation, Conceptualization, Methodology, Writing – review & editing. **Saverio Francini:** Conceptualization, Methodology, Writing – review & editing. **Costanza Borghi:** Writing – original draft. **Gherardo Chirici:** Conceptualization, Methodology, Writing – review & editing.

## Declaration of Competing Interest

The authors declare that they have no known competing financial interests or personal relationships that could have appeared to influence the work reported in this paper.
